# 超高效液相色谱-三重四极杆复合线性离子阱质谱法测定防脱发化妆品中13种酪氨酸激酶抑制剂

**DOI:** 10.3724/SP.J.1123.2025.02008

**Published:** 2025-10-08

**Authors:** Leilei FAN, Maoqin CHEN, Haibo WANG, Qiuhong YANG

**Affiliations:** 河南省药品医疗器械检验院（河南省疫苗批签中心），河南 郑州 450018; Henan Institute for Drug and Medical Device Inspection （Henan Vaccine Issuance Center），Zhengzhou 450018，China

**Keywords:** 防脱发化妆品, 超高效液相色谱-三重四极杆复合线性离子阱质谱, 酪氨酸激酶抑制剂, 非法添加, anti-alopecia cosmetics, ultra-high performance liquid chromatography-triple quadrupole composite linear ion trap mass spectrometry （UHPLC-Q-TRAP/MS）, janus kinase （JAK） inhibitors, illegally added

## Abstract

建立一种可准确、快速测定防脱发化妆品中13种酪氨酸激酶（janus kinase，JAK）抑制剂的超高效液相色谱-三重四极杆复合线性离子阱质谱检测方法。取适量样品，采用0.1%（v/v）甲酸水溶液-乙腈提取，乙腈提取液于‒20 ℃冷冻1 h，高速离心、过滤，采用C_18_色谱柱，在40 ℃的柱温下进行分离，以0.1%（v/v）甲酸水溶液-乙腈为流动相，梯度洗脱，流速0.3 mL/min；采用电喷雾正离子（ESI^+^）模式，多反应监测-信息关联采集-增强子离子（MRM-IDA-EPI）扫描方式，在高灵敏度分析的同时进行二级谱库检索，增加定性结果的准确性。在所设定的条件下，13种JAK抑制剂分离良好，并在所考察的范围内线性关系良好，*r*值均大于0.996。13种JAK抑制剂的检出限为1.5~1.7 ng/g，定量限为9.2~10.9 ng/g，在1倍、2倍和10倍定量限水平下进行加标回收试验，各待测物在水溶性基质化妆品中的平均回收率范围为94.7%~102.2%，RSD为2.2%~7.5%；膏霜乳类基质化妆品中的平均回收率范围为92.4%~99.2%，RSD为4.0%~8.8%。本检测方法准确、高效、操作简单，已应用到国家化妆品风险抽检工作中，为化妆品的监管提供技术支持。

## Abstract (en)

According to the Safety and Technical Specification for Cosmetics （2015）， the addition of chemical drugs to cosmetics is strictly prohibited. Anti-alopecia cosmetics are often found to contain illegal additions of prohibited drugs such as minoxidil， finasteride and other substances. Ultra-high performance liquid chromatography-tandem triple quadrupole-linear ion trap mass spectrometry （UHPLC-Q-TRAP/MS） has become a powerful technology for the simultaneous detection of illegal ingredients in cosmetics due to its advantages of rapid analysis， high sensitivity， high throughput and high selectivity. An ultra-high performance liquid chromatography-multiple reaction monitoring-information dependent acquisition-enhanced production scanning （UHPLC-MRM-IDA-EPI） method was developed to determine 13 JAK inhibitors in anti-alopecia cosmetics， including baricitinib， tofacitinib， ritlecitinib， peficitinib， abrocitinib， upadacitinib， ivarmacitinib， fedratinib， filgotinib， ruxolitinib， momelotinib， pacritinib and bozitinib. The influence of extraction solvents was investigated. Approximately 0.2 g of the sample was weighed and placed in a 50 mL graduated centrifuge tube with a cap. Then， 2 mL of 0.1% （v/v） aqueous formic acid and 10 mL of acetonitrile were added， followed by vortexing for 1 min. Subsequently， the mixture was sonicated in an ultrasonic bath for 15 min. Added 0.5 g of sodium chloride， and the mixture was centrifuged at 8 000 r/min for 8 min at 5 °C. The supernatant was transferred to another 50 mL graduated centrifuge tube with a cap. The residue was added with 10 mL of acetonitrile and the extraction procedure was repeated. After high-speed centrifugation， the supernatant was combined. The combined supernatant was added with 0.2 g of sodium chloride， and the mixture was frozen at ‒20 °C for 1 h. It was centrifuged at 8 000 r/min for 5 min at 5 °C. The acetonitrile layer was collected and filtered through a 0.22 μm organic filter membrane. The initial filtrate was discarded， and the subsequent filtrate was collected as the sample solution. A C_18 _column was used for chromatographic separation， enabling the successful separation of the analytes within 10 min. The mobile phase consisted of 0.1% （v/v） aqueous formic acid and acetonitrile， with gradient elution applied to optimize separation efficiency. The flow rate was set at 0.3 mL/min， and the column temperature was maintained at 40 ℃ to ensure consistent performance. A fixed injection volume of 2 μL was used to guarantee reproducibility and accuracy in the analysis. This configuration enabled the rapid and reliable separation of the target compounds. For the analysis of the 13 kinds of JAK inhibitors， an ESI source operating in positive ion mode was used with MRM-IDA-EPI detection. Quantification was performed by the external standard method using matrix-matched standard solutions. Good separation of the 13 JAK inhibitors was achieved under the optimized chromatographic conditions. Calibration curves were constructed by plotting the peak area of quantitative ions against the compound mass concentration. These curves exhibited excellent linearity within the investigated concentration range， with *r* all exceeding 0.996. The limits of detection （LODs） and limits of quantification （LOQs） of the 13 JAK inhibitors were 1.5**–**1.7 ng/g and 9.2**–**10.9 ng/g. To validate accuracy and precision， two cosmetic matrices （water-soluble and cream） were tested at three levels： LOQ， 2×LOQ and 10×LOQ. Recoveries of the 13 JAK inhibitors ranged from 94.7% to 102.2% for the water-soluble matrix and from 92.4% to 99.2% for the cream matrix， with relative standard deviations （RSDs） ≤8.8%. This method is characterized by high efficiency， rapidity， accuracy， sensitivity and simplicity， making it a powerful tool for rapid risk screening and simultaneous quantitative analysis of JAK inhibitors in anti-alopecia cosmetics.

斑秃脱发，影响个人外在形象，引发病人自卑心理，影响其日常工作和生活。结构性精神病学评估认为，全球1/3~3/4的斑秃病人均存在不同程度的精神合并征^［1］^。以前治疗斑秃脱发的主要药物有激素^［2］^、米诺地尔^［3］^、中药^［4］^等。章星琪^［5］^报道了酪氨酸激酶（janus kinase，JAK）抑制剂通过抑制Janus激酶-信号转导与转录激活因子通路，能有效抑制多种炎症因子，口服JAK抑制剂对斑秃脱发的有效治愈率可达80%，虽然JAK抑制剂外用疗效不如口服疗效好，但JAK抑制剂外用容易渗透，可作为斑秃脱发的辅助治疗。Xing等^［6］^、Bayart等^［7］^、李毓芬等^［8］^也报道了系统或局部外用JAK抑制剂能促进毛发再生。董亚蕾等^［9］^曾在防脱发化妆品中同时检出激素非那雄胺和米诺地尔两种药物，JAK抑制剂也可能会被非法添加在防脱发化妆品中。国内外有采用HPLC、HPLC-MS/MS方法测定血液、尿液或保健食品中托法替尼、帕克替尼、非戈替尼、巴瑞替尼、乌帕替尼、培非替尼等JAK抑制剂的分析报道^［10-15］^，但暂未发现在化妆品中检测JAK抑制剂的报道。

液相色谱-三重四极杆质谱检测因其灵敏度高、选择性好、抗基质干扰能力强，已被广泛应用于痕量成分和非法添加药物的检测^［16-18］^，但在实践中仍有假阳性误判情况的出现。采用超高效液相色谱-三重四极杆复合线性离子阱质谱（UHPLC-Q-TRAP/MS）进行多反应监测-信息关联采集-增强子离子扫描（MRM-IDA-EPI）检测^［19-21］^，可有效避免质谱检测过程中假阳性结果的出现。

针对在防脱发化妆品中可能非法添加13种JAK抑制剂（托法替尼、巴瑞替尼、利特昔替尼、鲁索替尼、培菲替尼、阿布昔替尼、乌帕替尼、艾玛昔替尼、菲达替尼、非戈替尼、莫洛替尼、帕克替尼、帕瑞替尼，其化学结构式见图1）的行为^［22-29］^，创建一个能准确、简便、快捷用于防脱发化妆品中非法添加JAK抑制剂检测的UHPLC-Q-TRAP/MS方法，为化妆品监管提供技术支持，具有现实意义。

**图1:**
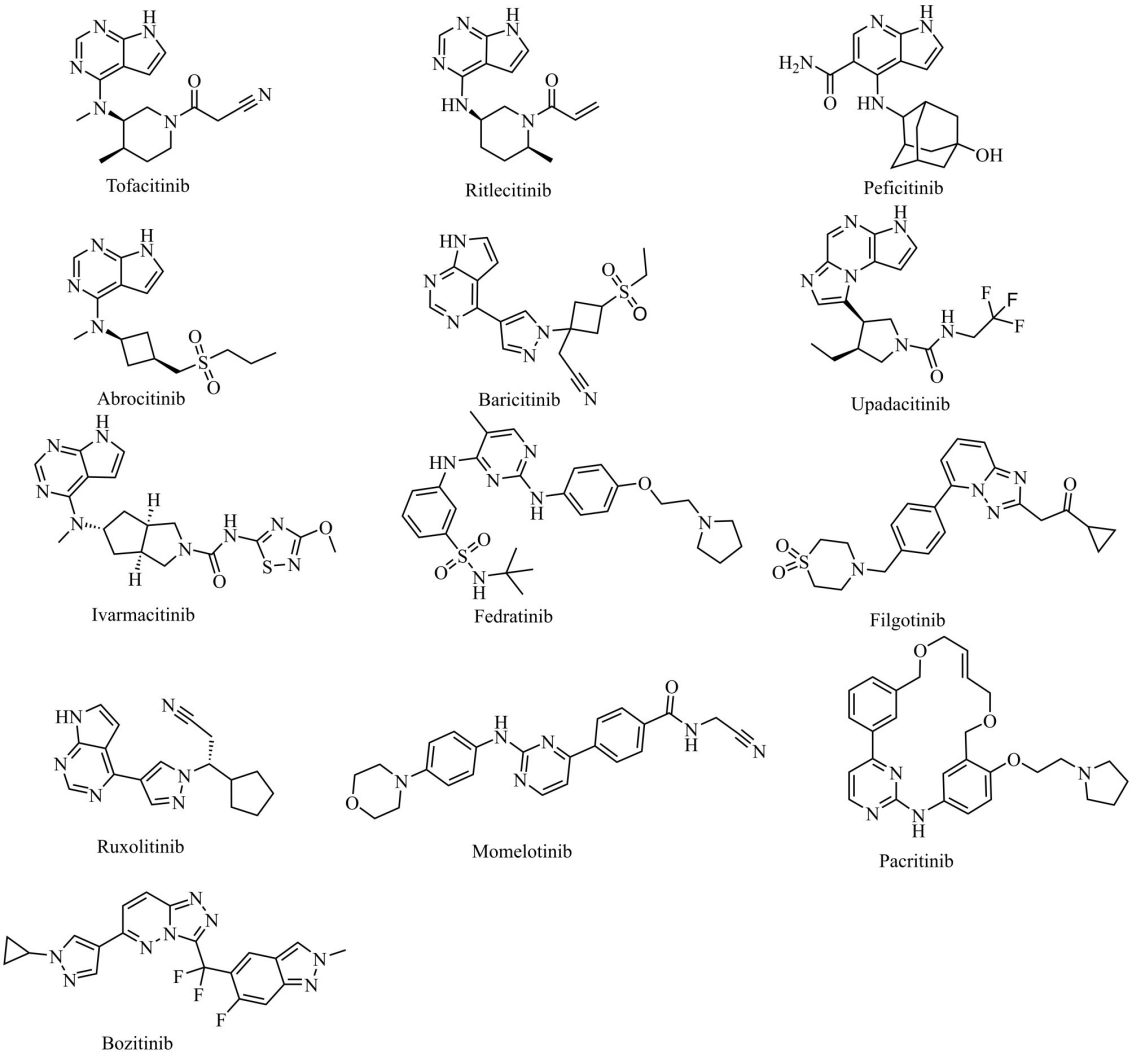
13种JAK抑制剂的化学结构式

## 1 实验部分

### 1.1 仪器、试剂与材料

AB ExionLC^TM^-Sciex Qtrap 6500+型超高效液相色谱**-**三重四极杆复合线性离子阱质谱仪（美国AB公司，配备Analyst^®^1.6.3数据采集软件、Multi Quant^TM ^3.0.2 software数据处理软件）；KQ-600DE型双频数控超声波清洗器（昆山市超声仪器有限公司）；3-30KS型高速离心机（德国Sigma公司）；MS3型旋涡混合仪（德国IKA公司）；Milli-Q超净纯水器（美国Millipore公司）；XPE 205型电子分析天平（瑞士Mettler公司，十万分之一精度）；2~20 μL、10~100 μL、20~200 μL、100~1 000 μL、0.5~5 mL Research plus型移液器（德国Eppendorf公司，配备无机溶剂、有机溶剂专用吸头，定期检定校准）。

13种JAK抑制剂均购自Stanford Chemicals公司，详细信息见表1；乙腈、甲醇（色谱纯，美国Merck公司）；甲酸、乙酸铵（质谱纯，比利时Acros公司）；Milli-Q超净纯水器制超纯水。56批宣称具有防脱发功效的化妆品均为常见的水溶性基质和膏霜乳类基质化妆品。

**表1:** 13种JAK抑制剂的CAS号、*M*_r_、分子式、批号、纯度及log *K*_ow_值

No.	Compound	Chinese name	CAS No.	*M*_r_	Molecular formula	Lot number	Purity/%	log *K*_ow_^*^
1	tofacitinib	托法替尼	477600-75-2	312.37	C_16_H_20_N_6_O	LR230627-04	99.90	1.83
2	ritlecitinib	利特昔替尼	1792180-81-4	285.34	C_15_H_19_N_5_O	TD230118-04	99.92	1.38
3	peficitinib	培菲替尼	944118-01-8	326.39	C_18_H_22_N_4_O_2_	BF230425-13	99.57	1.55
4	abrocitinib	阿布昔替尼	1622902-68-4	323.41	C_14_H_21_N_5_O_2_S	DB231227-13	99.92	1.94
5	baricitinib	巴瑞替尼	1187594-09-7	371.42	C_16_H_17_N_7_O_2_S	PL230609-17	99.55	‒0.58
6	upadacitinib	乌帕替尼	1310726-60-3	380.37	C_17_H_19_F_3_N_6_O	BD230918-17	99.51	2.71
7	ivarmacitinib	艾玛昔替尼	1445987-21-2	414.48	C_18_H_22_N_8_O_2_S	RT230430-12	98.20	4.01
8	fedratinib	菲达替尼	936091-26-8	524.68	C_27_H_36_N_6_O_3_S	TE230524-07	98.41	4.54
9	filgotinib	非戈替尼	1206161-97-8	425.50	C_21_H_23_N_5_O_3_S	BE231207-19	98.90	2.01
10	ruxolitinib	鲁索替尼	941678-49-5	306.37	C_17_H_18_N_6_	ML220916-02	99.93	2.09
11	momelotinib	莫洛替尼	1056634-68-4	414.46	C_23_H_22_N_6_O_2_	BR230807-11	98.22	2.38
12	pacritinib	帕克替尼	937272-79-2	472.58	C_28_H_32_N_4_O_3_	CR231104-02	99.70	3.68
13	bozitinib	帕瑞替尼	1440964-89-5	424.38	C_20_H_15_F_3_N_8_	SD231018-28	98.20	3.81

* EPI Suite^TM^ Version 4.11 estimation.

### 1.2 标准溶液配制

精密称取13种标准品适量，用70%乙腈水溶液分别制成储备溶液，储备溶液于4 ℃冰箱保存。

分别精密吸取13种单一标准品储备溶液各0.1 mL，混合放置于同一100 mL容量瓶中，用70%乙腈水溶液稀释定容至刻度，制得质量浓度约为2 μg/mL的混合标准品储备溶液。临用前，用新制备的空白基质（随机选取检测结果呈阴性的水溶性和膏霜乳类样品，作为空白基质）提取液稀释上述混合标准品储备溶液，制得所需浓度的基质匹配标准品工作溶液。

### 1.3 样品前处理

称取样品约0.2 g，放置于50 mL带盖刻度圆底塑料管中，先加入0.1%（v/v）甲酸水溶液2 mL、乙腈10 mL，涡旋混匀1 min，再置于超声振荡器中超声处理15 min，加入0.5 g氯化钠，5 ℃下高速（8 000 r/min）离心8 min，吸取上清液转移于另一50 mL带盖刻度圆底塑料管中，残渣再加入乙腈10 mL，重复萃取1次，高速（8 000 r/min）离心8 min，取上清液与第一次所得上清液合并，合并上清液中再加入0.2 g氯化钠，置于‒20 ℃冷冻1 h，取出后于5 ℃下高速（8 000 r/min）离心5 min，取乙腈层，用0.22 μm有机型滤膜过滤，弃去初滤液，取续滤液作为样品溶液（样品溶液可根据实际浓度用乙腈进行适当稀释）。

### 1.4 分析条件

采用色谱柱Waters CORTECS T3 C_18_（100 mm×2.1 mm，2.7 μm）；进样量：2 μL；柱温：40 ℃；流动相A：0.1%（v/v）甲酸水溶液，流动相B：乙腈；梯度程序洗脱：0~2.0 min，15%B；2.0~7.0 min，15%B~80%B；7.0~8.0 min，80%B~15%B；8.0~10.0 min，15%B；流速：0.3 mL/min。

电喷雾正离子（ESI^+^）模式下，MRM-IDA-EPI监测方式；离子源电压：+5 500 V；离子源温度：550 ℃；雾化脱溶剂气压：379 kPa；辅助加热气压：379 kPa；气帘气压：207 kPa；碰撞气能量等级：Medium；驻留时间15 ms。13种JAK抑制剂的保留时间、监测离子对、碰撞能量等MRM质谱参数详见表2。EPI模式下线性离子阱质谱参数：碰撞能量（CE）：50 eV，碰撞能散（CES）：20 eV。

**表2:** 13种JAK抑制剂的保留时间及相关MRM质谱参数

No.	Compound	*t*_R_/min	Parent ion （*m/z*）	Daughter ions （*m/z*）	CEs/eV
1	tofacitinib	1.66	313.2	149.1^*^， 173.2	39， 50
2	ritlecitinib	1.84	286.2	215.1^*^， 98.1	35， 34
3	peficitinib	1.95	327.2	160.1^*^， 310.2	52， 44
4	abrocitinib	2.24	324.1	149.1^*^， 134.1	34， 48
5	baricitinib	3.72	372.1	251.1^*^， 186.1	37， 45
6	upadacitinib	4.04	381.2	256.1^*^， 213.1	38， 55
7	ivarmacitinib	4.15	327.2	160.1^*^， 310.2	23， 50
8	fedratinib	4.45	525.3	469.3^*^， 97.9	39， 39
9	filgotinib	4.87	426.2	291.2^*^， 223.1	38， 55
10	ruxolitinib	5.34	307.1	131.0^*^， 159.2	46， 51
11	momelotinib	5.99	415.2	369.1^*^， 286.2	37， 60
12	pacritinib	6.19	473.3	316.2^*^， 95.9	43， 75
13	bozitinib	6.30	425.1	405.2^*^， 206.1	29， 62

CE： collision energy； * quantitative ion.

## 2 结果与讨论

### 2.1 提取条件优化

根据已有文献，Kumar等^［13］^、熊歆等^［14］^采用乙腈提取帕克替尼、巴瑞替尼；李慧玲等^［15］^采用甲醇提取托法替尼、非戈替尼、巴瑞替尼、培菲替尼、乌帕替尼；赵珊等^［30］^、丁晓静等^［31］^采用水-乙腈萃取盐析法^［32］^提取极性相差较大的化合物。取防脱发化妆品，分别加入13种JAK抑制剂标准物质，以回收率为参考指标，分别考察样品直接采用20 mL乙腈或甲醇超声提取，以及样品采用12 mL 0.1%（v/v）甲酸水溶液-乙腈=（2∶10，v/v）为溶剂的萃取盐析法提取等3种提取方式（见图2）。结果表明：甲醇在提取基质相对复杂的膏霜乳类化妆品中的13种JAK抑制剂时，回收率整体高于乙腈，但这与各化合物log *K*_ow_值显示的亲脂性较强，采用乙腈提取效果应该更佳的理论不符。分析出现此种现象的原因是乙腈提取基质相对复杂的膏霜乳类样品时，因乙腈具有很强的氢键接受能力，会强烈地与膏霜乳类样品中的水分子结合，争夺水分子，从而破坏膏霜乳类样品中固有的稳定胶体结构（主要是乳液和/或凝胶结构），使样品中原本被乳化剂分散的亲脂性成分（油脂、蜡质、某些疏水活性物、聚合物）失去水合环境，相互之间的疏水作用力增强，导致内相（油相或水相）组分、增稠剂、乳化剂等发生相分离、聚集、脱水收缩或凝胶收缩，导致局部黏度增加，阻碍乙腈的渗透和扩散，使其无法深入样品内部，导致目标物不能被充分提取出来。采用0.1%（v/v）甲酸水溶液-乙腈（2∶10，v/v）提取13种JAK抑制剂时，回收率最高，因此选为所用。

**图2:**
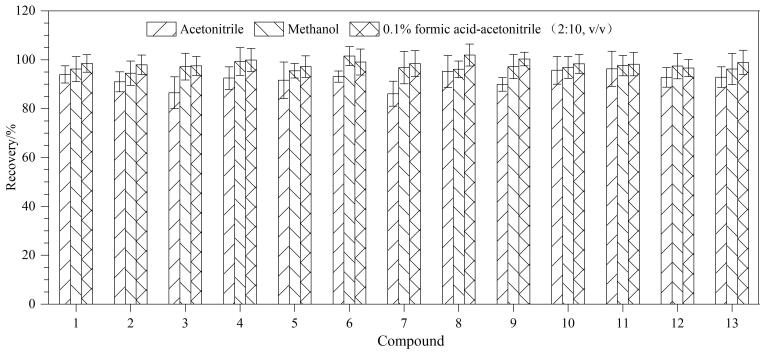
不同提取溶剂对膏霜乳类化妆品中13种JAK抑制剂回收率的影响 Compounds 1‒13 were the same as those in Table 1.

### 2.2 色谱条件优化

13种JAK抑制剂的极性范围差别较大，有些物质化学结构相似，且均含氨基，大部分具有疏水性，Kim等^［10］^、Dixit等^［11，12］^、Kumar等^［13］^、熊歆等^［14］^、李慧玲等^［15］^均采用C_18_色谱柱分离JAK抑制剂。Waters CORTECS T3 C_18_色谱柱既能增加强极性分子如含羟基、氨基等极性官能团的色谱柱保留，又能减少疏水性成分的保留，故选择Waters CORTECS T3 C_18_色谱柱进行试验。

Dixit等^［11，12］^、Kumar等^［13］^、熊歆等^［14］^、李慧玲等^［15］^采用液相色谱-质谱分析JAK抑制剂时，有机相采用乙腈或甲醇，水相采用低浓度的乙酸铵水溶液或甲酸水溶液。本研究选用乙腈和甲醇为有机相，选用纯水、0.2 mmol/L乙酸铵水溶液、0.1%（v/v）甲酸水溶液为水相，分别进行组合试验。结果表明，有机相选用乙腈时各色谱峰出峰时间较快；水相中加乙酸铵对各色谱峰的影响不大，采用0.1%（v/v）甲酸水溶液时，个别成分的色谱峰响应值比采用纯水时略高。故流动相最终选择乙腈和0.1%（v/v）甲酸水溶液。13种JAK抑制剂的MRM色谱图见图3。

**图3:**
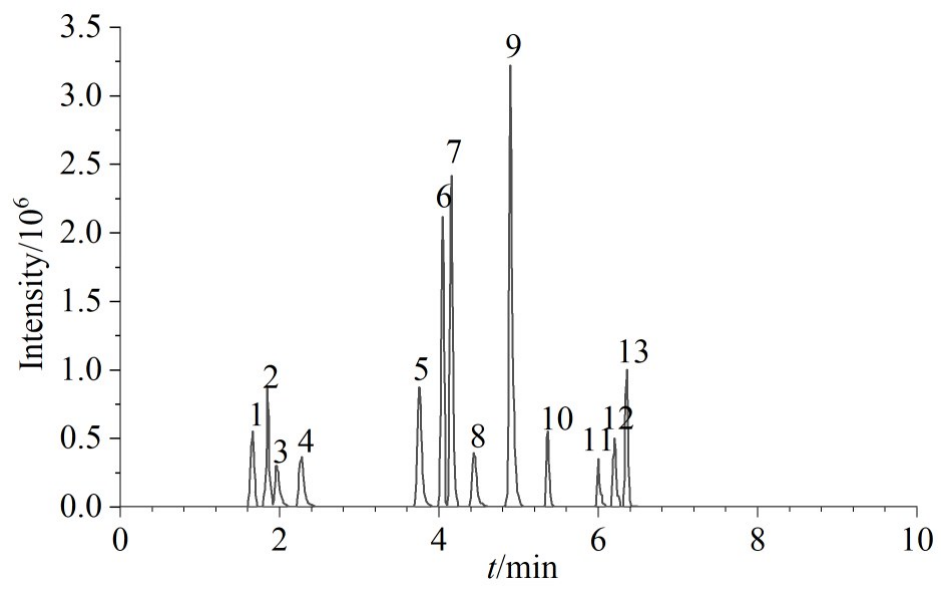
13种JAK抑制剂的MRM色谱图 Peaks 1‒13 were the same as those in Table 1.

### 2.3 质谱条件优化

取各成分的单标溶液，进行质谱监测参数的优化，结果各成分在正离子模式下的响应值较高，一级质谱获得分子离子作为母离子，二级质谱全扫描时，选择丰度响应值较高、周边干扰少且连续稳定出现的两个特征碎片离子作为子离子，选择其中响应较高的子离子作为定量离子。优化两个子离子的去簇电压、碰撞能量等，确定MRM质谱参数。结合流动相，进行质谱参数的微调，结果发现流动相中增加甲酸，部分成分的MRM色谱峰信号响应值会略有升高。MRM质谱条件确定后，结合二级质谱图信息的代表性，综合优化EPI模式下增强子离子扫描时的质谱参数，最终确定EPI质谱参数中的碰撞能量和碰撞能散。

按上述条件，取13种JAK抑制剂的标准品工作溶液，创建EPI二级谱库（见图4）。检出疑似阳性样品时，将样品分析时获得的EPI二级谱图与谱库进行匹配比对，相似程度越高，定性结果的准确度越高，避免假阳性误判情况的出现，保证检测结果的准确、公正、可信。

**图4:**
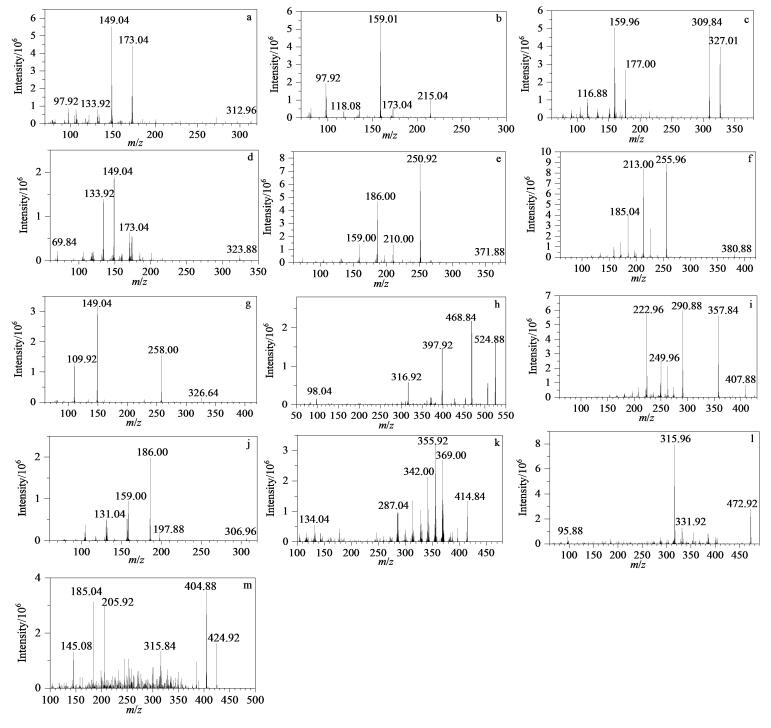
13种JAK抑制剂的增强子离子扫描二级质谱图 a. tofacitinib； b. ritlecitinib； c. peficitinib； d. abrocitinib； e. baricitinib； f. upadacitinib； g. ivarmacitinib； h. fedratinib； i. filgotinib； j. ruxolitinib； k. momelotinib； l. pacritinib； m. bozitinib.

### 2.4 基质效应考察

参考杨飘飘等^［33］^采用ME=基质配制标准品溶液曲线的斜率/溶剂配制标准品溶液曲线的斜率×100%的计算方法考察基质效应，若ME>100%，则基质对待测物存在增强效应；ME=100%，则说明基质效应影响不存在，对待测物提取影响可以忽略；若ME<100%，则为基质对待测物存在抑制效应。ME与100%之间的差值越大，表明基质对待测物存在的基质效应越明显。

实验考察了两种常见防脱发化妆品水溶性基质和膏霜乳类基质对13种JAK抑制剂检测的基质效应。结果表明：水溶性基质中各成分的ME值为87%~103%，膏霜乳类基质中各成分的ME值为83%~94%，两种基质对13种JAK抑制剂检测均存在少量不同程度的基质抑制效应，整体上水溶性基质对13种待测化合物质谱检测的基质抑制效应较膏霜乳类基质的影响弱，这与基质所含化学成分的种类不同有关。为了减少基质影响，保证分析定量计算结果的准确性，采用空白基质溶液稀释标准品溶液配制成系列相应基质匹配标准品工作溶液，进行外标法定量计算。

### 2.5 方法学考察

#### 2.5.1 线性范围、检出限、定量限

取混合标准品储备液，分别用水溶性基质和膏霜乳类基质防脱发化妆品的空白基质提取液制备不同质量浓度的系列基质匹配标准品工作溶液，进行分析测定。以系列基质匹配标准品工作溶液的质量浓度为横坐标（*x*，ng/mL），以定量离子对相应的峰面积为纵坐标（*y*），绘制标准曲线。13种JAK抑制剂在两种不同基质中均具有良好的线性关系，*r*均在0.996以上（见表3）。以混合标准品溶液采用空白基质提取液稀释至信噪比（*S*/*N*）为3时对应的含量为该方法的检出限（LOD），13种JAK抑制剂的LOD为1.5~1.7 ng/g；按国家药监局颁布的“化妆品补充检验方法研究起草技术指南”中定量限的定义要求，选择线性最低浓度水平点对应的含量为方法定量限（LOQ），13种JAK抑制剂的LOQ为9.2~10.9 ng/g。

**表3:** 13种JAK抑制剂在两种基质中的线性范围、检出限、定量限、线性方程和相关系数

Compound	Linear range/ （ng/mL）	LOD/ （ng/g）	LOQ/ （ng/g）	Water-soluble matrix		Cream matrix	
				Linear equation	*r*	Linear equation	*r*
Tofacitinib	1.07‒107.4	1.7	10.7	*y*=125766.1*x*+26790.7	0.9972	*y*=184568.6*x*+2225.7	0.9976
Ritlecitinib	1.09‒108.9	1.7	10.9	*y*=162599.2*x*+29019.2	0.9981	*y*=143265.2*x*+16012.2	0.9984
Peficitinib	0.92‒91.6	1.5	9.2	*y*=67432.9*x*+7472.9	0.9972	*y*=69871.5*x*+6631.7	0.9961
Abrocitinib	0.99‒99.4	1.6	9.9	*y*=93843.2*x*+20857.8	0.9983	*y*=82458.5*x*-1252.3	0.9988
Baricitinib	1.05‒105.1	1.7	10.5	*y*=176971.2*x*+33005.9	0.9985	*y*=185443.3*x*+43527.7	0.9976
Upadacitinib	1.00‒100.0	1.6	10.0	*y*=344171.1*x*+51436.1	0.9971	*y*=315487.2*x*+3825.7	0.9976
Ivarmacitinib	0.99‒98.9	1.6	9.9	*y*=346899.2*x*+63263.4	0.9982	*y*=317518.9*x*+35378.2	0.9965
Fedratinib	0.95‒95.0	1.5	9.5	*y*=70861.9*x*+151.5	0.9975	*y*=71454.2*x*-247.8	0.9975
Filgotinib	0.97‒97.5	1.6	9.7	*y*=519839.1*x*+70499.4	0.9974	*y*=620576.5*x*+9863.3	0.9979
Ruxolitinib	1.00‒100.4	1.6	10.0	*y*=151030.2*x*+35649.3	0.9966	*y*=135657.5*x*-12236.7	0.9961
Momelotinib	1.01‒100.8	1.6	10.1	*y*=30662.4*x*+4279.5	0.9972	*y*=35458.2*x*‒2212.8	0.9965
Pacritinib	0.99‒99.2	1.6	9.9	*y*=79112.0*x*‒1226.8	0.9963	*y*=75568.3*x*‒1053.7	0.9974
Bozitinib	0.94‒93.7	1.5	9.4	*y*=159928.2*x*+19241.3	0.9975	*y*=158218.5*x*‒3322.4	0.9969

*y*： peak area； *x*： mass concentration， ng/mL.

#### 2.5.2 回收率与精密度

选取水溶性基质和膏霜乳类基质两类防脱发化妆品作为空白加标基质，在13种JAK抑制剂的定量限、2倍定量限和10倍定量限3个水平下进行加标回收试验，每个水平平行试验6份，分析结果见表4。

**表4:** 两种基质中13种JAK抑制剂的回收率和RSD（*n*=6）

Compound	Spiked level/ （ng/g）	Water-soluble matrix		Cream matrix		Compound	Spiked level/ （ng/g）	Water-soluble matrix		Cream matrix	
		Recovery/%	RSD/%	Recovery/%	RSD/%			Recovery/%	RSD/%	Recovery/%	RSD/%
Tofacitinib	10.7	95	5.1	95	5.1	Fedratinib	9.5	94.7	6.4	94.7	8.8
	21.5	96.6	4	96.2	5.5		19	101.6	5.1	92.9	7.2
	107.4	99.4	5.2	97.9	7.9		95	102.2	4	96.1	8.5
Ritlecitinib	10.9	95.8	5.5	92.6	5.3	Filgotinib	9.7	98.2	5.4	93.2	7.5
	21.8	98.2	2.2	95.8	7.1		19.5	97.4	4.2	95.3	6.8
	108.9	98.8	3.4	93.7	7.6		97.5	99.7	3.8	98.7	6.2
Peficitinib	9.2	97.5	3.9	95.8	7.8	Ruxolitinib	10	96	6.3	94.4	8.3
	18.3	100	5	98.7	4.7		20.1	98	4.5	99.2	8.3
	91.6	99.6	4.2	97.4	6.2		100.4	98.1	5.1	96.1	7.3
Abrocitinib	9.9	96.4	5.3	94.7	7.4	Momelotinib	10.1	99.5	2.5	94.6	7.8
	19.9	98.9	5	96.8	7.4		20.2	96.7	5.5	95.4	7.1
	99.4	98.3	4.7	95.5	8.1		100.8	98.5	4.7	97.9	7.2
Baricitinib	10.5	97.3	5	92.4	6.2	Pacritinib	9.9	94.9	5.1	94.9	7.7
	21	97.7	2.9	93.6	5.1		19.8	100.3	4.6	97.4	8.5
	105.1	98.7	3.9	93.5	8.3		99.2	95.8	5.2	95.7	6.8
Upadacitinib	10	96.1	5.4	96.1	7.1	Bozitinib	9.4	98.5	3.3	97.7	7.1
	20	98.5	5	98.5	6.5		18.7	100.7	3.4	96.8	6
	100	97.5	3.7	97.7	7.4		93.7	99	7.5	98.4	4
Ivarmacitinib	9.9	95	3.9	94.2	6.7						
	19.8	98.7	4.3	97.5	5						
	98.9	98.2	5.1	97	7.1						

从表4可见，13种JAK抑制剂在不同水平下，水溶性基质化妆品平均回收率为94.7%~102.2%，RSD为2.2%~7.5%；膏霜乳类基质化妆品平均回收率为92.4%~99.2%，RSD为4.0%~8.8%。结果表明，所建立的防脱发化妆品中13种JAK抑制剂的检测方法准确、可靠，可用于此类化妆品的检验检测。

### 2.6 实际样品测定

采用所建立的方法对56批防脱发或生发育发类化妆品进行检测分析，暂未检出上述13种JAK抑制剂，但采用此方法进行的化妆品安全风险监测筛查工作仍在持续之中。作为国家化妆品抽检安全风险监测的探索性研究方法，本方法的建立为化妆品的风险监测和高通量筛查提供了技术支撑，有利于加强化妆品安全监管。

## 3 结论

本实验建立了采用超高效液相色谱**-**三重四极杆复合线性离子阱质谱同时测定巴瑞替尼等13种JAK抑制剂的方法。该方法具有准确、简便、快速、专属性强等优势，通过一次进样同时满足高灵敏度的定量分析和基于EPI谱库的定性筛查需求，可以有效避免检测结果“假阳性”情况的出现，为防脱发类化妆品的非法添加检测提供新的检验方法，对企图在化妆品中非法添加此类药物的不法分子起到震慑作用，同时也为化妆品的安全监管提供技术支持，该方法已应用于2024年国家化妆品抽检工作。
